# The preservation of bidirectional promoter architecture in eukaryotes: what is the driving force?

**DOI:** 10.1186/1752-0509-6-S1-S21

**Published:** 2012-07-16

**Authors:** Chao Xu, Jiajia Chen, Bairong Shen

**Affiliations:** 1 Center for Systems Biology, Soochow University, Suzhou 215006, China

## Abstract

**Background:**

The bidirectional gene architecture has been studied in many organisms, and the conservation of bidirectional arrangement has received considerable attention. However, the explanation for the evolutionary conservation about this genomic structure is still insufficient. In this study the large scale identification and pathway enrichment analysis for bidirectional genes were performed in several eukaryotes and the comparative analysis of this arrangement between human and mouse were dissected for the purpose of discovering the driving force of the preservation of this genomic structure.

**Results:**

We identified the bidirectional gene pairs in eight different species and found this structure to be prevalent in eukaryotes. The pathway enrichment analysis indicated the bidirectional genes at the genome level are conserved in certain pathways, such as the DNA repair and some other fundamental cellular pathways. The comparative analysis about the gene expression, function, between human and mouse bidirectional genes were also performed and we observed that the selective force of this architecture doesn't derive from the co-regulation between paired genes, but the functional bias of bidirectional genes at whole genome level is observed strengthened during evolution.

**Conclusions:**

Our result validated the coexpression of bidirectional genes; however failed to support their functional relevance. The conservation of bidirectional promoters seems not the result of functional connection between paired genes, but the functional bias at whole genome level, which imply that the genome-wide functional constraint is important for the conservation of bidirectional structure.

## Background

The bidirectional promoters, as a special arrangement of neighbouring genes, have been discussed in many previous studies. The bidirectional gene pairs were defined as the divergent genes with the distance between their transcription start sites (TSS) less than 1 kb [1]. The frequency distribution of distance between adjacent gene pairs showed that the bidirectional promoters are prevalent in human genome [1]. It was later discovered that this genomic architecture is also abundant in mouse, Arabidopsis thaliana, yeast and many other species [2-4]. Comparative genomic analysis suggested that this gene-pair structure is conserved in vertebrates [2,5,6]. It was therefore believed that the bidirectional promoters possess special biological meaning [2-4,7].

The co-regulation was believed to be the distinctive feature of bidirectional gene pairs, and the mechanism of the similarity of expression profiles may be the sharing of the regulatory elements [1]. The previous study by Li *et al *concluded that this genomic arrangement is ancient and conserved during the evolutionary process, where the function relevance of this structure was also reported in the literature [5]. Other comparative genomic researches about the bidirectional gene pairs were also performed [2,6], but the reason for the structure conservation is still not clear now. The comparative analyses about the expression and function attribution of bidirectional gene pairs at whole genome level between human and mouse in our work could provide the potential explanations for this question.

In this study, we first performed the large-scale identification and pathway enrichment analysis of bidirectional gene pairs among several eukaryotes. Then we analyzed the general evolutionary tendency of this architecture. The functional preference of bidirectional genes at whole genome level was discovered and this preference was found to be conserved among species. The function relevance at the paired genes level as the driving force for the preservation of bidirectional promoters was excluded. The functional bias of bidirectional genes at the whole genome level is strengthened in human compared with mouse, which may imply the genuine origin of the conservation of bidirectional architecture.

## Results

### Bidirectional promoters are prevalent in eukaryotic genomes

**Figure 1:**
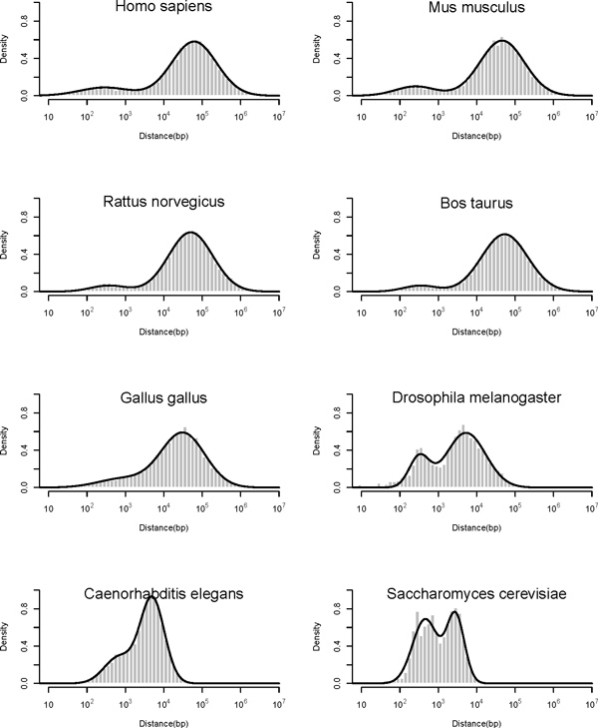
**Distribution of distance between head to head gene pairs in eight eukaryotic organisms**. The distances between TSSs of head to head gene pairs were calculated in eight organisms individually. The distance distribution curves were then smoothed using Kernel Density Estimation. The binomial distribution can be observed in all the eight charts, where the minor peak represents the enrichment of bidirectional promoters.

**Figure 2:**
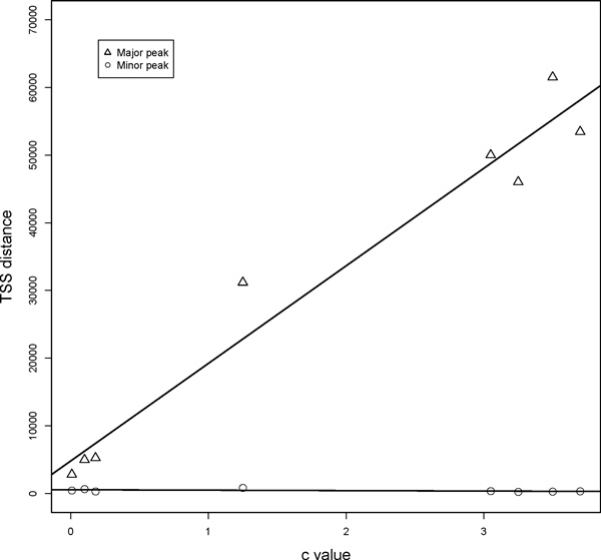
**Relationship between percentage of bidirectional genes and genome c-value**. The percentages of bidirectional genes were computed in all eight organisms. The percentages were fitted with corresponding genome c-value using linear regression fitting. The negative correlation was observed between percentage of bidirectional genes and genome c-value.

The bimodal distribution for the distance between transcription start sites (TSSs) of adjacent genes in opposite strands was detected in human genome, and the minor peak was considered as the peak of the distance between bidirectional gene pairs [1]. The distribution of distance between TSSs of neighbouring genes on opposite strands in eight individual eukaryotes was summarized, and the Kernel Density Estimation [8] was then applied to smooth the histograms with Gaussian curves (Figure 1). Then we fitted the distance distribution with two mixed Gaussian distributions and obtained the approximate locations of the minor and major peaks (Additional file 1). The fitting result reveals that the locations of minor peaks are more stable among species and maintain less than 1 kb while the location of major peaks tends to be proportional to the genome size. The Pearson correlation coefficient reflects that the significant correlation between the predicted location of major peak and the genome c-value (R^2 ^= 0.9578, p-value = 2.379E-05), however the correlation was not observed in the minor peak (p-value = 0.185 (Figure 2). The c-value of each genome was extracted from the Animal Genome Size Database [9]. Although the genome size varies among species, the location of minor peak possesses considerable stability which on the other hand confirms the identification criteria of distance between TSSs less than 1kb for defining bidirectional promoters in former research [1].

The bidirectional promoters were then identified via employing the criteria of TSSs distance less than 1 kb in eight eukaryotic organisms. The number of recognized bidirectional promoters and the bidirectional genes are shown in Table 1. Kanako (2005) argued that the enrichment of bidirectional pairs was not observed in non-mammals [2]. However, even though the minor peaks in some species are not that obvious in the distance distribution, the percentage of bidirectional genes is still considerable, and the percentage goes up along with the decreasing genome size (Table 1). As a result, the bidirectional arrangement should be prevalent in eukaryotic genomes. It has been raised that there is a negative relationship between the ratio of bidirectional genes and the gene density of each chromosome in human and mouse genome [5]. This correlation can also been discovered across species when comparing the ratio of bidirectional genes with the genome size (Pearson correlation test, R^2 ^= 0.547, p-value = 0.036). Interestingly, the percentage of bidirectional genes falls sharply from invertebrates to vertebrates. This may be the consequence of the large-scale segmental duplications which was believed to increase gene numbers and genome size during the origin of vertebrates [10].

**Table 1:** Statistical results of bidirectional promoters and bidirectional genes in the eight selected eukaryotes.

Organism	Number of bidirectional promoters	Number of bidirectional genes	Number of all protein coding genes	Percentage of bidirectional genes
**Homo sapiens**	1178	2348	20686	11.35%
**Mus musculus**	1311	2617	22793	11.48%
**Rattus norvegicus**	698	1393	22925	6.08%
**Bos taurus**	574	1144	19030	6.01%
**Gallus gallus**	687	1367	15310	8.93%
**Drosophila melanogaster**	2210	4398	13671	32.17%
**Caenorhabditis elegans**	1813	3612	20212	17.87%
**Saccharomyces cerevisiae**	1864	3423	6664	51.37%

The statistical results about the bidirectional pairs and involved genes, the genome c-value for the eight organisms were also enclosed in the table.

The prevalence of bidirectional promoters indicates this genomic architecture or the involved genes may have some special properties which make them preserved during the evolutionary history. We attempted to provide a potential explanation by the comparative analysis of bidirectional promoter among species, especially between human and mouse genome.

### Co-regulation of bidirectional gene pairs hardly determines the fate of bidirectional promoters

It was examined that the sequence of bidirectional promoters can regulate both divergent genes [1]. As a result, the co-regulation of paired bidirectional genes can be expected. The co-expression level of paired bidirectional genes had been confirmed to be significantly higher than other neighbouring gene structures by whole-genome microarray data analysis [1,5]. The significant function relevance had also been observed in the paired genes [5].

However, there are two potential deficiencies in the former analyses. First, the tandem duplications, which may cause the co-regulation of paired gene as trivial reasons [11], must be removed to purify the influence of bidirectional promoters. The tandem duplications, representing the genes duplicated in tandem [12], have pretty high sequence similarity and show symmetry not only in gene expression but also in function. Second, the similar expression pattern in neighbouring genes has been reported in human, drosophila and C. elegans [11,13,14], and chromatin-level gene regulation are thought as the most probable explanation for this phenomenon [11]. Consequently, in order to exclude the contribution of chromatin-level gene regulation, the co-regulation level of bidirectional genes should be compared with other adjacent gene architectures as well as the random gene pairs.

**Figure 3:**
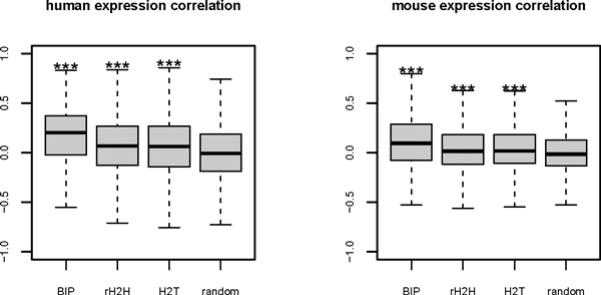
**Pearson correlation coefficient for four kinds of gene pairs in human and mouse genome**. The Pearson correlation coefficient between the expression profiles of paired genes in the four categories of gene pairs: BIP, rH2H, H2T and random pairs were computed. The *** represents that the correlation coefficient for this pair was significantly higher than random pairs (p-value < 0.01).

The neighbouring gene pairs in human genome were divided into three classes: 1) bidirectional gene pairs (BIP), which represent the adjacent gene pairs with TSSs distance less than 1 kb on the opposite strands; 2) remote head to head gene pairs (rH2H), the adjacent gene pairs on the opposite strands except the bidirectional gene pairs; 3) head to tail gene pairs (H2T), which represent the neighbouring gene pairs on the same strands. The tandem duplications were excluded from all these three architectures, and the randomly paired genes were chosen as the control. The Pearson correlation coefficient between gene expression profiles was computed employing the wide-distributed microarray data across 78 human cell types and 62 mouse cell types stemmed from a previous transcriptome analysis by Su *et al *[15]. A significant high correlation was observed when comparing the co-expression level of all adjacent genes pairs with the random pairs (Figure 3; Wilcoxon test P-value < 2.2E-16). Meanwhile, the bidirectional gene pairs had significant higher coexpression level than the rH2H and H2T (Wilcoxon test, P-value 1.139E-12, 1.225E-13). This analysis was also conducted in the mouse genome, and the results agreed with that of human genome (Figure 3). After excluding the tandem duplications and considering the influence of local expression similarity, the bidirectional genes still possess similar expression profiles. The shared control region may be the most reasonable explanations for this coordinated expression.

**Figure 4:**
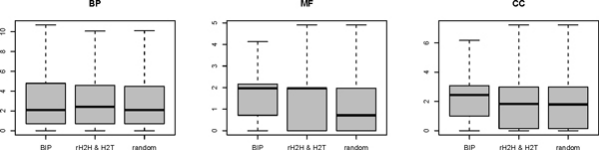
**The functional similarities for human bidirectional genes**. The box plot for the bidirectional gene pairs in three GO subsystems "biological process"(BP), "molecular function"(MF), "cellular component"(CC) reflects that the similarity in BP term was not obvious.

The functional similarity of bidirectional gene pairs has been evaluated in a former study [5], and the significant correlation were found in all three Gene Ontology(GO) subsystems. The functional similarity between genes was quantified as the Resnik probability. Here we applied the same GO Resnik semantic measure described in Li *et al*'s literature [5], for the "biological process" (BP) no significant co-function tendency was observed comparing with the random gene pairs after excluding the tandem duplications. As for the subsystem "molecular function" (MF), there is a significant higher functional similarity compared with random gene pairs (p-value = 0.007345), however this discrepancy was not found when compared with other adjacent gene pairs. Consistent with Li's result, the tendency to show functional relevant in subsystem "cellular component" (CC) is stronger not only compared with random pair (p-value = 5.877E-13) but also with other neighbouring gene pairs (p-value = 1.469E-07) (Figure 4). In general, the convinced functional relationship of bidirectional genes can only be found in the cellular component term. Among the three GO subsystems, the BP refers to the biological objective to which the gene or gene product contributes [16], and this term preferably represents the biological function of the gene. In consideration of this fact, the functional relevance may not be the statistically significant attribution for bidirectional genes. Moreover, we examined the bidirectional gene pairs involving DNA repair. The bidirectional genes have been found to be enriched in DNA repair pathways [1,7]. The DNA repair genes were collected by the pathway annotation in KEGG pathway. For the 105 DNA repair genes, 35 are regulated by bidirectional promoter; however, all the 35 paired genes don't perform a role in DNA repair.

As pointed out by Yanai and his co-worker, the similar gene expression profiles may not imply similar functions [17], so the co-expression of bidirectional genes may not be driven by the biological function but by the shared regulatory elements. The co-regulation of bidirectional gene pairs may not serve as the selection criteria of this genome architecture but the consequence.

### The function preference of bidirectional genes increase along with the selection of bidirectional architecture

It was reported that the genes involved in DNA-repair are more likely to be arranged in bidirectional manner [1,7,18]. The functional preference of bidirectional genes deserves considerable attention. Here we adopted the hypergeometric distribution to find the enriched KEGG pathway for bidirectional genes as described in Materials and Methods. The enriched pathways of bidirectional genes in eight selected organisms were shown in Table 2. There are some enriched function classes which are constant among species, especially the transcription and transcription related (Splicesome, RNA-degradation, RNA polymerase) and DNA-repair-related pathways (Nucleotide excision repair, Non-homologous end-joining). It was pointed out that the bidirectional gene pairs are only conserved in vertebrates, the bidirectional linkages are disorganized during the evolution from invertebrates to the vertebrates [2]. Nevertheless, the enriched pathways among all the eukaryotes show substantial conservation. Although the bidirectional linkages of paired genes were lost during the evolution, some genes in particular pathways were still regulated by bidirectional promoters but paired with another gene. The genome greatly expanded at the origin of vertebrates, but otherwise remained relatively constant [19]. As a result, the bidirectional linkages in invertebrates were broken and shuffled at the emerging of vertebrates which cause the non-conservation of bidirectional gene pairs between invertebrates and vertebrates.

**Table 2:** Enriched KEGG pathways of bidirectional genes in eight different species.

*Pathway*	*human*	*mouse*	*rat*	*cow*	*chicken*	*fruit fly*	*C.elegans*	*yeast*	*Count*
**Spliceosome**	hsa03040	mmu03040	rno03040	bta03040	gga03040	dre03040	cel03040	sce03040	8
**RNA degradation**	hsa03018	mmu03018	rno03020	bta03018		dre03018	cel03018	sce03018	7
**Nucleotide excision repair**	hsa03420	mmu03420	rno03420		gga03420	dre03420	cel03420	sce03420	7
**RNA polymerase**	hsa03020	mmu03020		bta03020		dre03020	cel03030	sce03020	6
**Non-homologous end-joining**	hsa03450		rno03450	bta03450	gga03450	dre03450	cel03450		6
**Oxidative phosphorylation**	hsa00190	mmu00190	rno00190	bta00190	gga00190		cel00190		6
**Pyrimidine metabolism**	hsa00240	mmu00240				dre00240	cel00240	sce00240	5
**Ribosome**	hsa03010			bta03010		dre03010	cel03010	sce03010	5
**Base excision repair**	hsa03410			bta03410	gga03410	dre03410		sce03410	5
**Purine metabolism**		mmu00230				dre00230	cel00230	sce00230	4
**DNA replication**	hsa03030	mmu03030				dre03030		sce03030	4
**Proteasome**					gga03050	dre03050	cel03050	sce03050	4
**Mismatch repair**	hsa03430					dre03430	cel03430	sce03430	4
**Homologous recombination**	hsa03440	mmu03440				dre03440	cel03440		4
**Aminoacyl-tRNA biosynthesis**	hsa00970		rno00970		gga00970		cel00970		4
**N-Glycan biosynthesis**		mmu00510	rno00510				cel00510		3
**Cell cycle**	hsa04110	mmu04110	rno04110						3
**Peroxisome**	hsa04146	mmu04146	rno04146						3
**Systemic lupus erythematosus**	hsa05322	mmu05322		bta05322					3
**O-Mannosyl glycan biosynthesis**		mmu00514			gga00514			sce00514	3
**Folate biosynthesis**						dre00790	cel00790	sce00970	3
**Metabolic pathways(Global Pathway)**	hsa01100						cel01100	sce01100	3
**Protein export**						dre03060	cel03060	sce03060	3
**Basal transcription factors**						dre03022		sce03022	2
**SNARE interactions in vesicular transport**						dre04130		sce04130	2
**Parkinson's disease**	hsa05012			bta05012					2
**Citrate cycle (TCA cycle)**			rno00020				cel00020		2

The first three letters represent the abbreviation of each organism and the following numbers mean the accession number of the pathway in KEGG. The COUNT is the number of species which is enriched with the pathway. The pathways which only enriched in one organism were excluded.

The more interesting finding is that the conserved bidirectional gene enriched pathways are more likely to involve the basic functions in cell. In order to confirm this observation, the tissue specificity of gene expression was then evaluated by the gene expression profiles. Large-scale gene expression variation has been used to select house-keeping genes in many former researches; the genes with lower expression variation among tissues are regarded as potential house-keeping genes [12,20,21]. The calculation formula for gene expression specificity is presented in Materials and Methods [22]. The tissue specificity of bidirectional genes is significantly lower than other genes (Wilcoxom sum rank test, p-value 2.459E-13 for human and 2.338E-13 for mouse), which means the bidirectional genes express widely among different tissues and prefer to perform fundamental functions.

In order to check the potential constraints for the conservation of bidirectional arrangements, we classified the human bidirectional genes into two categories: the conserved bidirectional genes whose mouse orthologous genes are still arranged in bidirectional architecture (human cBIP gene) and the human-specific bidirectional genes whose mouse orthologous genes are not regulated by bidirectional promoter (human sBIP gene). The human-mouse one to one orthologous gene pairs were extracted from Ensembl using Biomart [23]. Only those bidirectional gene pairs with one to one orthologous mouse genes were discussed in this study. The mouse bidirectional genes were also classified based on this criterion. The enriched pathways of cBIP genes show great similarity in human and mouse, all the enriched pathways in mouse are also enriched in human genome, which indicates the function classes of preserved bidirectional genes are stable during evolution. However, the pathway enrichment for sBIP genes varies widely between human and mouse (Table 3, Table 4). The above observations imply that the bidirectional genes tend to perform functions in particular pathways and this tendency can determine the selective conservation of bidirectional gene pairs. For instance, the DNA-repair related pathways can be found in human cBIP genes (hsa03410, hsa03420, hsa03430), mouse cBIP genes (mmu03420, mmu03430) and human sBIP genes (hsa03410, hsa03420), but not in mouse sBIP genes. The genes which do not perform the DNA-repair functions were eliminated from the bidirectional arrangement during the evolution of human genome structure, while the co-opted bidirectional genes in human genome more and more participate in these pathways. As a conclusion, the bidirectional genes tend to perform particular fundamental functions like DNA repair and this function preference may affect the fate of bidirectional structure during evolution; however the reason for the trend of enrichment in these particular pathways need further investigation.

**Table 3:** Enriched KEGG pathway of human cBIP and sBIP gene classes.

KEGG pathway ID	Pathway Name	p-value	gene type
hsa03040*	Spliceosome	4.24E-04	cBIP
hsa03020*	RNA polymerase	1.09E-03	cBIP
**hsa05012**	Parkinson's disease	1.64E-03	cBIP
**hsa05010**	Alzheimer's disease	1.78E-03	cBIP
**hsa00190**	Oxidative phosphorylation	1.93E-03	cBIP
hsa03018*	RNA degradation	1.93E-03	cBIP
**hsa04110**	Cell cycle	3.01E-03	cBIP
**hsa04142**	Lysosome	4.32E-03	cBIP
**hsa05016**	Huntington's disease	5.11E-03	cBIP
**hsa00970**	Aminoacyl-tRNA biosynthesis	8.60E-03	cBIP
**hsa00052**	Galactose metabolism	1.54E-02	cBIP
**hsa03440**^**#**^	Homologous recombination	2.10E-02	cBIP
**hsa04146**	Peroxisome	3.62E-02	cBIP
**hsa03420**^**#**^	Nucleotide excision repair	4.03E-02	cBIP
**hsa00230**	Purine metabolism	4.32E-02	cBIP
**hsa03410**^**#**^	Base excision repair	4.47E-02	cBIP
**hsa00240**	Pyrimidine metabolism	2.28E-02	sBIP
**hsa00562**	Inositol phosphate metabolism	2.36E-02	sBIP
**hsa01100**	Metabolic pathways	9.65E-03	sBIP
hsa03020*	RNA polymerase	9.05E-03	sBIP
**hsa03030**	DNA replication	1.92E-02	sBIP
**hsa03050**	Proteasome	4.85E-02	sBIP
**hsa03410**^**#**^	Base excision repair	1.58E-02	sBIP
**hsa03420**^**#**^	Nucleotide excision repair	3.70E-02	sBIP
**hsa04115**	p53 signaling pathway	4.81E-02	sBIP
**hsa05215**	Prostate cancer	4.30E-02	sBIP

The two categeries of human bidirectional genes (cBIP, sBIP) were computed for the KEGG pathway enrichment respectively. cBIP represents the genes regulated by the human-mouse conserved bidirectional promoters, while the sBIP represents the genes in the species-specific bidirectional gene pairs. * Transcription and translation related pathways. # DNA repair related pathways

**Table 4:** Enriched KEGG pathway of mouse cBIP and sBIP gene classes.

KEGG pathway ID	Pathway Name	p-value	gene type
mmu03040*	Spliceosome	3.30E-03	cBIP
**mmu04110**	Cell cycle	3.70E-03	cBIP
**mmu04142**	Lysosome	4.13E-03	cBIP
mmu03018*	RNA degradation	5.70E-03	cBIP
**mmu00970**	Aminoacyl-tRNA biosynthesis	8.74E-03	cBIP
mmu03020*	RNA polymerase	8.97E-03	cBIP
**mmu00190**	Oxidative phosphorylation	1.07E-02	cBIP
**mmu03440**^**#**^	Homologous recombination	1.36E-02	cBIP
**mmu00052**	Galactose metabolism	1.59E-02	cBIP
**mmu05012**	Parkinson's disease	1.98E-02	cBIP
**mmu04146**	Peroxisome	3.03E-02	cBIP
**mmu03420**^**#**^	Nucleotide excision repair	3.30E-02	cBIP
**mmu00240**	Pyrimidine metabolism	6.22E-03	sBIP
mmu03020*	RNA polymerase	5.15E-03	sBIP
**mmu00062**	Fatty acid elongation in mitochondria	1.10E-02	sBIP
**mmu00903**	Limonene and pinene degradation	1.40E-02	sBIP
**mmu00280**	Valine, leucine and isoleucine degradation	1.98E-02	sBIP
**mmu05340**	Primary immunodeficiency	4.08E-02	sBIP
**mmu00860**	Porphyrin and chlorophyll metabolism	4.96E-02	sBIP

The two catteries of mouse bidirectional genes (cBIP, sBIP) were computed for the KEGG pathway enrichment respectively. cBIP represents the genes regulated by the human-mouse conserved bidirectional promoters, while the sBIP represents the genes in the species-specific bidirectional gene pairs. * Transcription and translation related pathways # DNA repair related pathways

## Discussion

In this study, we found that the bidirectional gene pairs were prevalent in eukaryotes and the percentage of bidirectional genes declines along with the increasing of genome size. The increasing of genome size is much faster than that of gene number during evolution, which can be attributed to two reasons. First, the growing number and length of introns make the gene longer [24]. Second, the intergenic distance is also greatly expanded. The expanding of intergenic distance inevitably reduces the percentage of bidirectional promoters.

Only protein-coding gene was considered in this work, the non coding transcripts which are pervasive in many organisms were recently found enriched in the upstream of protein-coding genes and shared the same promoter with the adjacent genes [4,25,26]. But most of the pervasive non-coding transcripts at bidirectional promoters were considered as unstable which would be degraded soon after the birth, and the function of them was not clear right now [27]. In an attempt to validate the distance distribution of head to head transcript pairs, we took the listed non-coding genes in Ensembl into consideration and re-identified the bidirectional promoters. Although this gene collection don't include all transcripts, the distribution is similar with the former (Additional file 2), and the minor peak which represents the bidirectional promoters indeed increased. However these transcripts are rarely expression quantified and function annotated. Our research focused on the evolution force of the efficient bidirectional promoters, so only the bidirectional promoters which encode two protein-coding genes were considered.

The co-regulation of bidirectional gene pairs has been reported in many studies including the co-expression and function relevance [1,5]. Here we validated the coexpression of bidirectional genes rather than the cofunction. The bidirectionality has been proved to be an inherent feature of promoters [1], and the proposed divergent transcription model also thought the genes were transcribed in both direction synchronously [27]. However the GO similarity analysis didn't agree with the functional relevance of paired genes. The bidirectional genes transcribed simultaneously but perform different function in cell. The co-regulation of bidirectional pairs may stem from the shared promoter; however it hardly has effect on the selection of bidirectional promoters because the natural selection of gene order bases on the functional relevance such as operon in prokaryotes. The shuffling of bidirectional linkage between invertebrates and vertebrates also proves the bidirectional structures are not kept by co-regulation.

The cross-species pathway enrichment analysis showed that the functions of bidirectional genes are greatly conserved in certain fundamental function classes like DNA-repair and transcription related pathways. And this function preference may increase along with the selection of bidirectional structure. The bidirectionality is the inherent feature of promoters [1], the < 1 kb interval between head to head gene pairs can basically determine the co-regulation of paired genes. We assumed that the surrounding nucleotide composition of these genes may be the genuine trigger, the upstream genome structure of these genes are more stable and avoid the insertion of non coding DNAs or other genes which leads to the shorter interval between adjacent gene; however this assumption requires further validation.

## Methods

### Identification of bidirectional promoters in eight eukaryote genomes

The chromosomal positions and sequences information of all the protein-coding genes were fetched from the Ensembl database [28] (Ensembl gene Build 58) using the Biomart system [29] for eight selected organisms: Homo sapiens, Mus musculus, Rattus norvegicus, Bos Taurus, Gallus gallus, Drosophila melanogaster, Caenorhabditis elegans and Saccharomyces cerevisiae. The mitochondria genome and unmapped fragments were not included in the following analysis. The gene start sites in Ensembl gene annotation database were regarded as the reliable transcription start site (TSS) of each gene because the full-length cDNA was used to confirm the gene boundaries [30]. The protein-coding genes on each chromosome were sorted according to the TSS coordinates. The neighbouring genes on the same strand were recognized as the head to tail gene pairs, while the opposite strand as the head to head gene pairs. Then the distances between head to head gene TSSs were calculated for the eight organisms respectively.

### Removal of tandem duplication

As indicated in the previous works, the tandem duplication can contribute to the local similarity of gene attributions and this substantially affects the neighbouring gene effect analysis [31]. We therefore removed the tandem duplications from the neighbouring gene pairs for the following coexpression and cofunction analysis. For each adjacent gene pairs, corresponding protein sequences were obtained from Ensembl database (Build 58), and then the protein sequences were imported into pair-wise BLAST to get the e-value of sequence similarity (standard setting, word size 2). This method with 0.2 as cut off value has been proved to be powerful to remove ~90% of related genes from a dataset [12]. In this article, we used smaller cut off to reduce false positive rate. The pair with e-value < 0.01 was regarded as tandem duplication and eliminated in the following gene pair similarity analysis.

### Extraction of conserved and species-specific bidirectional gene pairs by orthologous linkage between human and mouse

If the human paired bidirectional genes both have the one-to-one orthologous gene in mouse genome and the orthologous gene pairs were still arranged in bidirectional architecture, these bidirectional gene pairs were counted as the conserved bidirectional gene pairs (human cBIP pairs), while other gene pairs as the human specific bidirectional gene pairs (human sBIP pairs). Similarly, the mouse bidirectional gene pairs are also divided into mouse cBIP pairs and sBIP pairs using the human-mouse linkage. The 14024 one-to-one orthologous gene relationships between human and mouse were extracted from Ensembl database via the Biomart. As a result, 540 human conserved bidirectional promoters and 270 human unique bidirectional promoters were classified, while these numbers are 540 and 207 in mouse genome.

### Pathway enrichment analysis of bidirectional genes

The KEGG database [32] collected the pathway information for many organisms, and we further determined if these pathways are enriched with the bidirectional genes using hypergeometric distribution. For a given pathway in a particular organism, we fixed the total number of protein-coding genes in this organism (N), the number of genes involving this pathway (N1), the number of total bidirectional genes in this organism (N2), and treated the number of bidirectional genes in this pathway as a random variable. Under the null hypothesis that the genes are not enriched in this pathway, this random variable follows a hypergeometric distribution. The enrichment p-value can be then defined as the probability that the gene number in this pathway is greater than or equal to the observed value (N0), which can be represented by the following equation:

$$\text{P}=1-\overset{N_{0}-1}{\underset{}{\sum }}\frac{\left(\begin{array}{c} {\text{N}}_{1}\\ \text{i} \end{array}\right)\left(\begin{array}{c} \text{N}-{\text{N}}_{1}\\ {\text{N}}_{2}-\text{i} \end{array}\right)}{\left(\begin{array}{c} \text{N}\\ {\text{N}}_{2} \end{array}\right)}$$

The calculations in the parentheses refer to the combinatorial calculation. Pathway was recognized as enriched with bidirectional genes if the p-value was lower than 0.05.

### Gene expression specificity and coexpression level

The raw microarray data were obtained from Su *et al. *[15]. For human genome 156 Affymatrix U133A microarray experiments across 78 human cell types were deployed, while for the mouse genome, the object of analysis turned into the 122 custom-designed GNF microarray chips representing 61 mouse cell types in Su's dataset. The microarray data was pre-processed by RMA method [33] with R affy package [34]. If a gene can map to several probesets, the mean value of the probesets' expression level was regarded as the gene expression value. For each gene the expression specificity was then calculated as the following equation:

$$\tau =\frac{\sum_{\text{i}=1}^{\text{n}}\left(1-\frac{{\text{E}}_{\text{i}}}{{\text{E}}_{\text{max}}}\right)}{\text{n}-1},$$

where n represents the number of expression datasets, Emax as the maximum expression value of all across cell type expression values, Ei as gene expression value in each microarray experiment. In human and mouse genome, for the mapped head to head gene pairs, head to tail pairs and random-generated 20000 gene pairs, the gene coexpression level were then evaluated as the Pearson correlation coefficient between expression profiles of paired genes separately.

### Gene Ontology association analysis

The GO annotation for each gene was extracted from Gene Ontology database [16]. For one gene, the direct annotation was extended to general annotation by appending all the parent nodes of the direct annotation in the GO vocabulary tree [5]. The detail about the algorithm of Resnik semantic similarity was discussed in Li's work [5]. Among all the neighbouring gene pairs, the functional similarities of annotated pairs were then calculated in all three GO subsystems: "biological process", "molecular function", "cellular component", employing an R package for computing semantic similarity based on Gene Ontology annotations called csbl.go [35].

## Competing interests

The authors declare that they have no competing interests.

## Authors' contributions

CX carried out the computational analysis, CX, JC and BS participated in the design and drafted the manuscript. BS conceived and coordinated this study. All authors read and approved the final manuscript.

## Supplementary Material

Additional file 1 **Predicted peaks of distribution of distance between TSSs of head to head gene pairs using two mixed Gaussian distribution**. The approximate positions of the minor and major peaks in the distance distributions were fitted in all the eight organisms. The average value for the minor peak and major peak were computed afterwards. Click here for file

Additional file 2 **Distribution of distance between genes and adjacent genes on opposite strand in human when including the non-coding transcripts**. a). The distribution when only considering protein-coding genes. b). The distribution when including non-coding genes. The non-coding transcripts were defined as the genes labelled by 'lincRNA', 'miRNA', 'miscRNA', 'rRNA', 'snoRNA', 'snRNA', 'non-coding', 'processed_transcript' in biotype term in Ensembl Build 58. Click here for file
