# Supplementary material for: The preservation of bidirectional promoter architecture in eukaryotes: what is the driving force?
Source: BMC Syst Biol. 2012 Jul 16;6(Suppl 1):S21. doi: 10.1186/1752-0509-6-S1-S21 (PMC3403606; doi:10.1186/1752-0509-6-S1-S21)
Supplement: Additional file 1 — Predicted peaks of distribution of distance between TSSs of head to head gene pairs using two mixed Gaussian distribution. The approximate positions of the minor and major peaks in the distance distributions were fitted in all the eight organisms. The average value for the minor peak and major peak were computed afterwards. [file 1752-0509-6-S1-S21-S1.pdf]

TableS1 - Predicted peaks of distribution of distance between TSSs of head to head gene pairs using two mixed Gaussian distribution.

The approximate positions of the minor and major peaks in the distance distributions were fitted in all the eight organisms. The average value for the minor peak and major peak were computed afterwards.

Organism	minor peak	major peak	C-value
Homo sapiens	291	61518	3.50
Mus musculus	256	46026	3.25
Rattus norvegicus	366	50003	3.05
Bos taurus	339	53456	3.70
Gallus gallus	838	31189	1.25
Drosophila melanogaster	318	5260	0.18
Caenorhabditis elegans	667	4989	0.10
Saccharomyces cerevisiae	457	2805	0.008
