# Supplementary figures and images for: The preservation of bidirectional promoter architecture in eukaryotes: what is the driving force?
Source: BMC Syst Biol. 2012 Jul 16;6(Suppl 1):S21. doi: 10.1186/1752-0509-6-S1-S21 (PMC3403606; doi:10.1186/1752-0509-6-S1-S21)

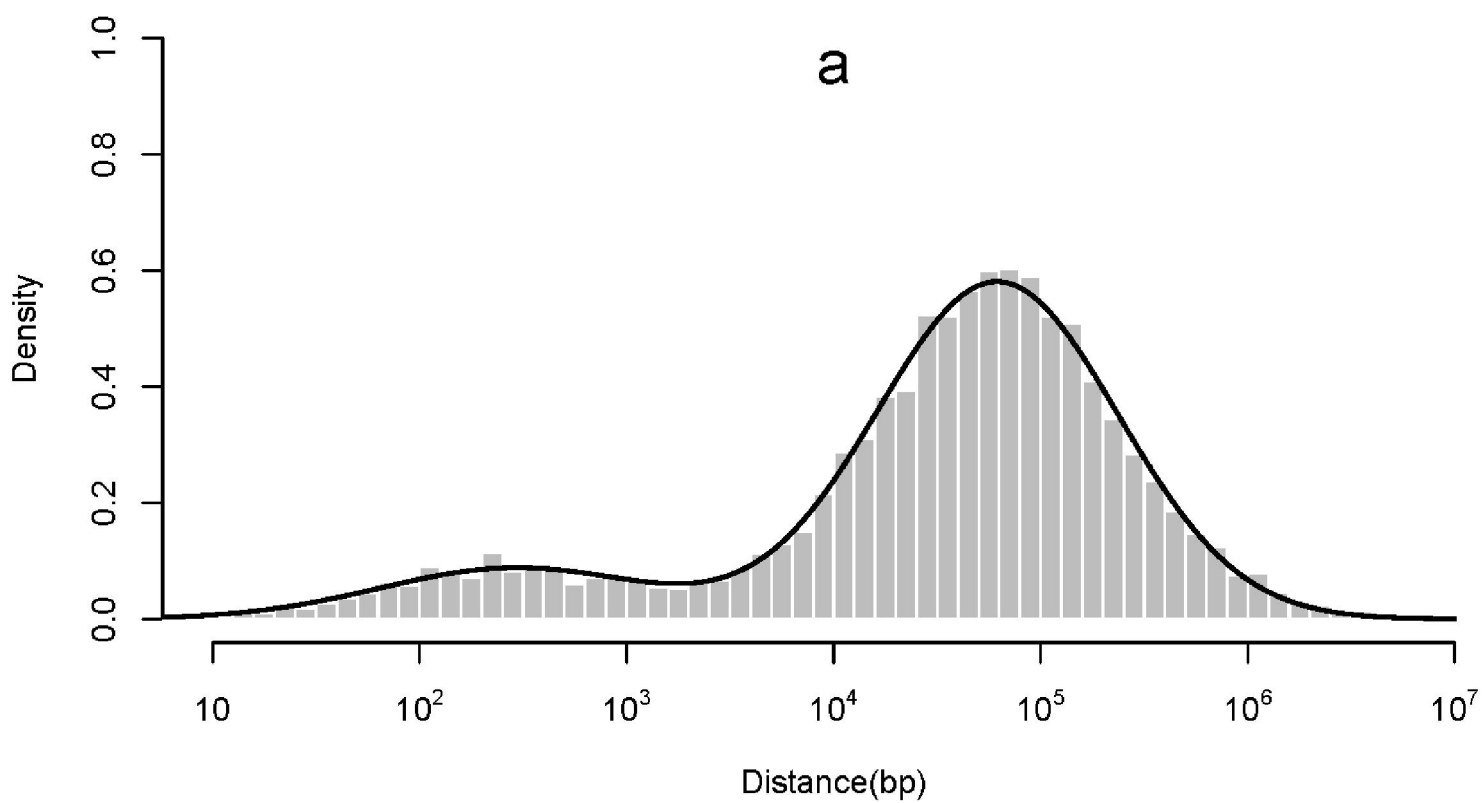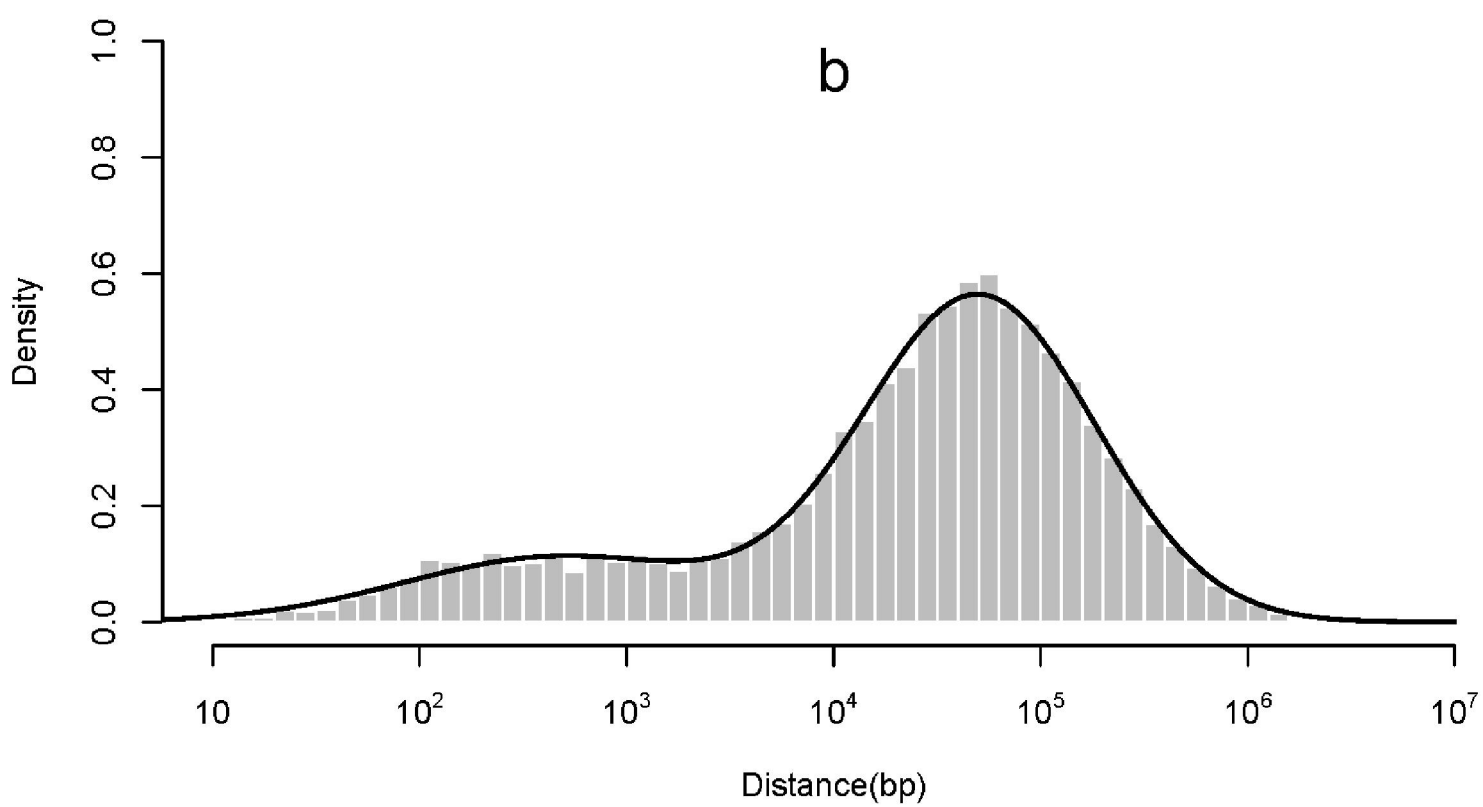

Supplement: Additional file 2 — Distribution of distance between genes and adjacent genes on opposite strand in human when including the non-coding transcripts. a). The distribution when only considering protein-coding genes. b). The distribution when including non-coding genes. The non-coding transcripts were defined as the genes labelled by 'lincRNA', 'miRNA', 'miscRNA', 'rRNA', 'snoRNA', 'snRNA', 'non-coding', 'processed_transcript' in biotype term in Ensembl Build 58. [file 1752-0509-6-S1-S21-S2.pdf]
